# Correction to “Vaccine Hesitancy or Hesitancies? A Latent Class Analysis of Pediatric Patients’ Parents”

**DOI:** 10.1111/cts.70277

**Published:** 2025-06-18

Willis DE, Narcisse MR, James L, Selig JP, Ason M, Scott AJ, Cornett LE, McElfish PA. Vaccine Hesitancy or Hesitancies? A Latent Class Analysis of Pediatric Patients’ Parents. Clinical and Translational Science. 2025;18(1):e70042. https://doi.org/10.1111/cts.70042.

In the article cited above, the correct grant number for the Community Engagement Alliance (CEAL) Against COVID‐19 Disparities was missing.

This part of the funding statement should have read, “This work is supported by the Community Engagement Alliance (CEAL) Against COVID‐19 Disparities (NIH 10T2HL156812‐01 and OT2HL158287)…”

We apologize for this error.

